# Inability to Ambulate in a Pediatric Patient Secondary to an Aneurysmal Bone Cyst With Associated Pathological Fracture

**DOI:** 10.7759/cureus.39310

**Published:** 2023-05-21

**Authors:** Halee Sowinski, Amber Vozar, Sara Demircan, Seth J Deskins, Sharda Udassi

**Affiliations:** 1 Pediatrics, West Virginia University School of Medicine, Morgantown, USA; 2 Internal Medicine-Pediatrics, West Virginia University School of Medicine, Morgantown, USA

**Keywords:** fractures, pediatric fractures, radiology, ortho surgery, pediatrics, bone cyst

## Abstract

Bone pain in pediatric patients is exceedingly common, with etiologies ranging from benign lesions such as fibrous dysplasia and enchondromas to potentially devastating, life-threatening malignancies such as Ewing's sarcoma or osteosarcoma. Given the low yield of physical examination and routine laboratory workup, pediatric patients with bone pain or an inability to ambulate warrant further workup. The initial workup should consist of imaging with radiography. A large majority of patients will have a resolution of symptoms without intervention and will have normal imaging. When radiographic imaging has suspicious findings, expert consultation is warranted because differentiating between benign and malignant processes on imaging can be challenging. Here we present a case of a six-year-old male with progressive worsening leg pain who was found to have a rare aneurysmal bone cyst with an associated pathologic fracture requiring open reduction and internal fixation.

## Introduction

Bone cysts are benign lesions most commonly found in the pediatric population during the second decade of life. These lytic lesions can be subclassified as unicameral or aneurysmal. Aneurysmal bone cysts (ABCs) are exceptionally rare, at a rate of 0.14 per 100,00 per year. Morphologically, aneurysmal bone cysts are expansive and hemorrhagic indeterminate bone tumors of intermediate malignancy, typically located at the metaphysis of long bones. On radiographic imaging, ABCs appear as eccentric, radiolucent, and expansile lesions containing cystic cavities that cause cortical bone bulges. Primary lesions have a genetic rearrangement of the USP6 oncogene on chromosome 17, but ABCs can be secondary to other bone lesions. A biopsy is essential to differentiate from telangiectatic osteosarcoma. Treatment includes wide resection to cure and prevent reoccurrences, osteosynthesis with graft, and cement or bone substitute for fragilization or pathological fracture.

## Case presentation

**Figure 1:**
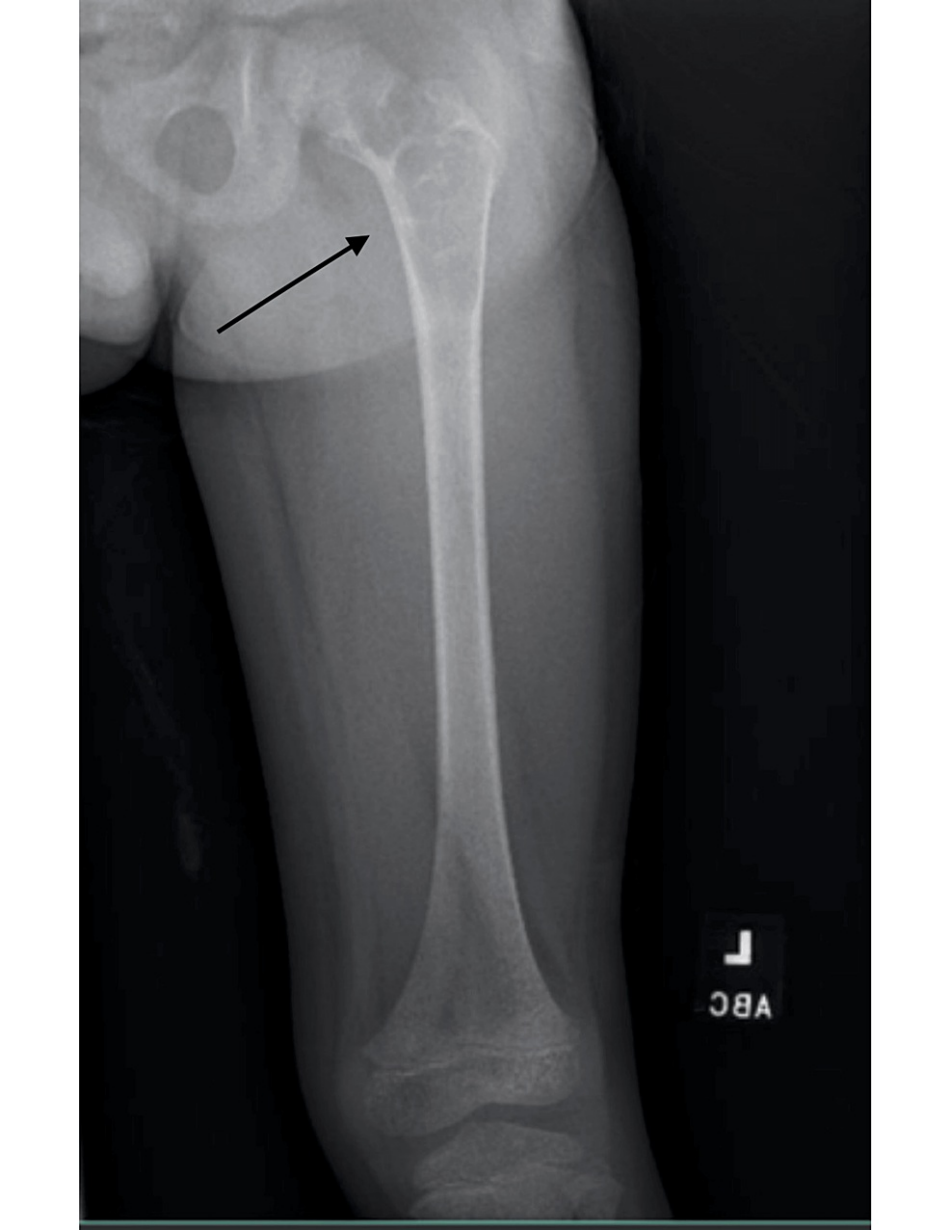
Radiograph showing an ill-defined lytic lesion of the left proximal femur with concern for pathologic fracture at the location of the arrow

A six-year-old male began having left leg pain in July 2022. His pediatrician took a radiograph of his knee and lower leg, which did not reveal any fracture or deformity. Over the span of a few months, his pain worsened to a limp when he attempted to play football, and he could no longer bear weight. In October 2022, the patient returned to his pediatrician due to his inability to ambulate. A repeat radiograph of the hip revealed an ill-defined lytic lesion with pseudotrabeculation involving the proximal left femoral diaphysis extending into the femoral. Along with this, there was a lucency involving the inferior portion of the neck of the femur, which likely represents a pathologic fracture (Figure 1). Given these findings and concern for underlying malignancy, the patient was transferred to a tertiary care center for further workup and pediatric orthopedic surgery consultation. On admission, routine laboratory workups, including a complete blood count and basic metabolic panel, were within normal limits. On physical exam, the lower left extremity showed no gross deformity, and motor movement and sensation to light touch were intact. Strength testing was 5/5 for knee extension and 4/5 for knee flexion. The patient’s pain was located on the mid-anterior left thigh, and he was unable to perform a straight leg raise. 

**Figure 2:**
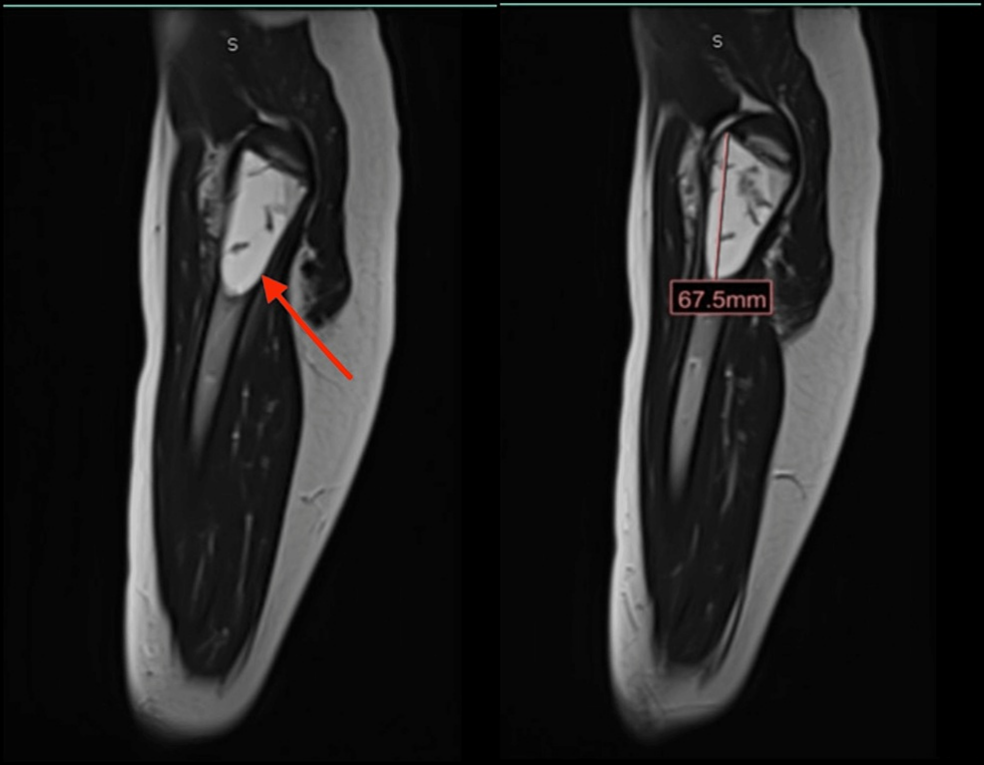
MRI showing a multiloculated expansile lesion of the left femoral neck with areas of layering hemorrhage and pathologic fracture

Orthopedic surgery recommended magnetic resonance imaging to further characterize the lesion (Figure 2).

**Figure 3:**
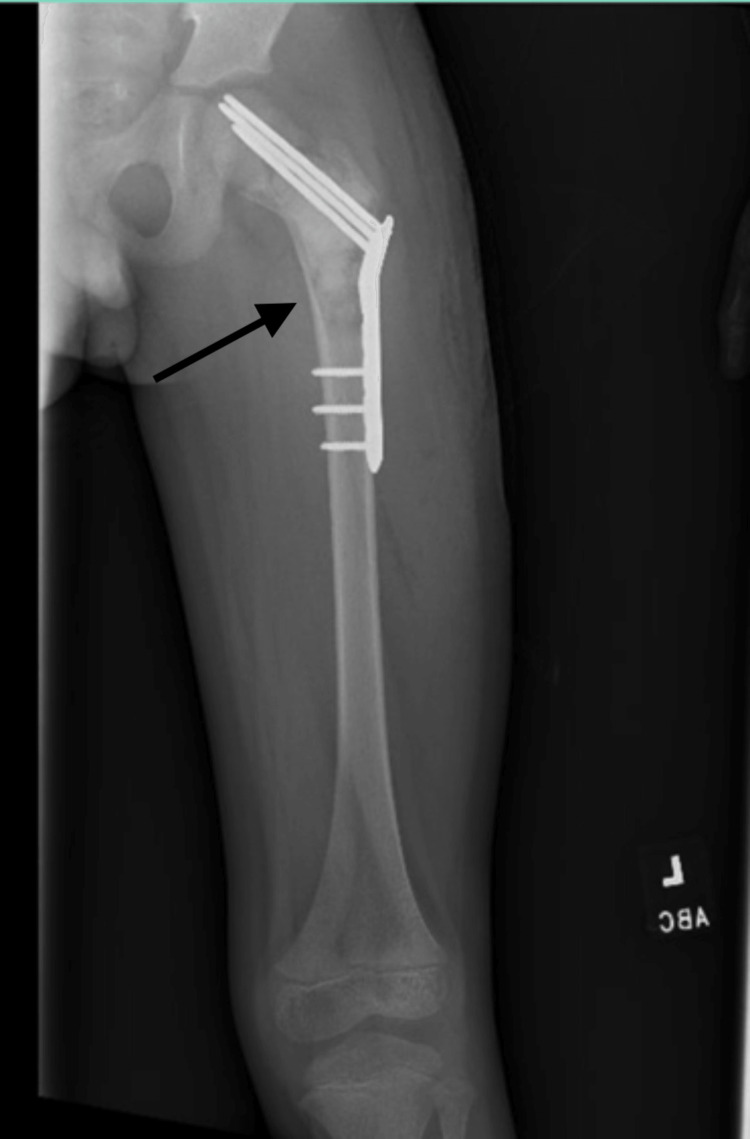
Radiograph after open reduction and internal fixation of a pathologic fracture and curettage of an aneurysmal bone cyst

Pediatric orthopedic surgery biopsied the cystic lesion, and pathology revealed benign-appearing cystic tissue. An open reduction and internal fixation with pins and a plate were completed for stabilization of the pathologic fracture (Figure 3).

Curettage of the cyst was performed, and the cystic space was filled with an injectable inductive graft and crushed cancellous bone. Physical therapy and occupational therapy provided education for ambulation with a walker upon discharge post-operative day one. Hematology/oncology followed the permanent pathology report, which ultimately diagnosed an aneurysmal bone cyst. 

## Discussion

Pediatricians often encounter patients with lower extremity pain in their practice. When a child presents with lower extremity pain, the initial assessment begins with a history and physical examination to evaluate the nature and timing of the pain, its impact on ambulation, and if any associated symptoms ensued in the same period. The physician assesses the joints for a range of motion, signs of infection, deformity, and gait, along with a neurovascular exam. The further investigation includes basic screening labs to screen for infectious etiology and radiographs of the affected site [1]. In our case, the only remarkable study was the patient's imaging, which revealed a suspicious lesion. Pain in the hip is often referred to as pain from the knee. When a patient presents with lower extremity pain and an unexplained limp, evaluation of both the knee and hip is crucial for diagnosis. Referred pain in the knee is often missed and can lead to a delay in the treatment of hip disease [2].

Young, well-appearing children with acute gait disturbance often self-resolve. In a study of 1- to 5-year-old outpatient children that presented for acute gait disturbance, radiographic studies appeared normal 96% of the time. In follow-up observations, 81% returned to their pediatrician within 4-28 months, all of which showed spontaneous resolution [3]. Additionally, a longitudinal radiographic review of asymptomatic children revealed an 18.9% prevalence of benign childhood bone tumors of the extremities [4]. Although limping may statistically self-resolve and bone tumors appear asymptomatically in approximately 1/5th of children, investigation for the cause of pain is increasingly important for the pediatric population due to its highly variable presentation. Evaluation of adjacent structures with limb pain is a crucial aspect of pediatric musculoskeletal evaluation [1]. Young children are not developmentally able to characterize the nature of pain and pinpoint the source of it, often leading to delays in actual diagnosis. Radiological imaging guidelines for a child with a mass involving bone suggest a radiograph of the entire bone containing the lesion, and urgent evaluation by a surgical specialist or oncologist is strongly recommended [5]. Magnetic resonance imaging is often used to better characterize a suspicious mass and provide more data for specialists when weighing observation versus surgical procedures. Furthermore, pediatric patients with acute limb pain, fever, overlying erythema, pinpoint pain or tenderness, and weight loss warrant referral to a specialist [4].

## Conclusions

Evaluation of a patient with a physical exam revealing moderate-to-severe pain and pinpoint tenderness over the anterior thigh should prompt initial imaging with radiography and consultation with orthopedic surgery if a suspicious lesion is found. When followed in the outpatient setting, the large majority of pediatric patients presenting with acute gait abnormalities will have normal imaging, and nearly all patients will have a resolution of symptoms. Regardless, key signs and symptoms such as pain or an inability to ambulate should prompt imaging and a referral to a specialist. Here we present a case of an aneurysmal bone cyst with an associated pathological fracture in a patient with an inability to ambulate.
